# Crystal Structure of Phosphoserine BlaC from *Mycobacterium tuberculosis* Inactivated by Bis(Benzoyl) Phosphate

**DOI:** 10.3390/ijms20133247

**Published:** 2019-07-02

**Authors:** Timothy W. Moural, Dawanna Shar-Day White, Cindy J. Choy, Chulhee Kang, Clifford E. Berkman

**Affiliations:** 1 Department of Chemistry, Washington State University, Pullman, WA 99164, USA; 2 Department of Entomology, Pennsylvania State University, University Park, PA 16802, USA

**Keywords:** *Mycobacterium tuberculosis*, β-lactam antibiotic resistance, β-lactamase, phosphorylation, crystal structure

## Abstract

*Mycobacterium tuberculosis*, the pathogen responsible for tuberculosis (TB), is the leading cause of death from infectious disease worldwide. The class A serine β-lactamase BlaC confers *Mycobacterium tuberculosis* resistance to conventional β-lactam antibiotics. As the primary mechanism of bacterial resistance to β-lactam antibiotics, the expression of a β-lactamase by *Mycobacterium tuberculosis* results in hydrolysis of the β-lactam ring and deactivation of these antibiotics. In this study, we conducted protein X-ray crystallographic analysis of the inactivation of BlaC, upon exposure to the inhibitor bis(benzoyl) phosphate. Crystal structure data confirms that serine β-lactamase is phosphorylated at the catalytic serine residue (Ser-70) by this phosphate-based inactivator. This new crystallographic evidence suggests a mechanism for phosphorylation of BlaC inhibition by bis(benzoyl) phosphate over acylation. Additionally, we confirmed that bis(benzoyl) phosphate inactivated BlaC in a time-dependent manner.

## 1. Introduction

According to the 2017 World Health Organization’s (WHO) report, *Mycobacterium tuberculosis*, the pathogen responsible for tuberculosis (TB), is the leading cause of death from infectious disease worldwide [1]. In 2016 alone, there were more than 1.6 million deaths linked to TB infection. High incidences of new infections are reported annually, with 6.1 million new cases in 2015 and 6.3 million reported in 2016. In addition, there are growing threats of emerging multidrug-resistant (MDR-TB) and extensively drug resistant (XDR-TB) strains of *Mycobacterium tuberculosis* that are resistant to the current first-line, second-line, and third-line drugs used to treat TB [1,2]. The WHO estimates 4.1% of new TB infections and 19% of those previously treated were caused by MDR-TB strains in 2016 [1].

With the emergence of MDR-TB and XDR-TB, novel research efforts are being focused on identifying new drug targets, such as enzymes of the nucleotide biosynthesis pathways and the TCA cycle [3]. Multiple novel antitubercular drugs are currently in the discovery phase and under clinical development [4]. One possible course of action to treat TB is to include β-lactam antibiotics to the list of agents used to treat TB infections [2,5]. Despite the successful use of β-lactam antibiotics to treat gram-negative and gram-positive bacterial infections over the last century, β-lactam antibiotics have not been commonly used to treat TB due to the expression of BlaC, a β-lactamase capable of hydrolyzing their β-lactam ring [2,6].

β-lactamases are categorized into four classes based on molecular characteristics, including sequence and structural similarities [6]. These four classes are A, B, C, and D, which can be classified into two main mechanistic groups. Class B β-lactamases are zinc metalloenzymes, while class A, C, and D β-lactamases are serine β-lactamases [6,7]. BlaC is a class A β-lactamase and due to its broad substrate specificity towards β-lactams, β-lactam antibiotics alone are not an efficacious treatment course for TB infections [8]. However, *Mtb* has demonstrated increased susceptibility to β-lactam antibiotics upon the inactivation of BlaC, thus making BlaC an important target for therapeutic agents [9,10].

With the emergence of pathogenic bacteria strains exhibiting broad-spectrum antibiotic resistance, it has been suggested that using a β-lactamase inhibitor in conjunction with a β-lactam antibiotic, could increase the likelihood of positive treatment outcomes [8,11]. To this point, β-lactam antibiotics have been used in conjunction with β-lactamase inhibitors as part of a multi-drug treatment regime for TB infections [2,11]. A number of studies have been undertaken to understand the evolution, enzyme structures, and catalytic mechanisms of various β-lactamases including BlaC [2,7,8,12,13]. Li and Pratt showed that acyl phosphonate scaffolds could be used to inhibit serine β-lactamases [14,15]. Herein, we provide crystal structure evidence to demonstrate that the serine β-lactamase BlaC can be phosphorylated at its nucleophilic serine by the novel β-lactamase inhibitors based on a bis(benzoyl) phosphate scaffold. These bis(benzoyl) phosphates are hypothesized to behave like traditional organophosphorylating agents that target serine hydrolases similar to acetylcholinesterase.

## 2. Results

### 2.1. Crystal Structures of Phosphoserine BlaC and Inactivation by Bis(Benzoyl) Phosphate

**Figure 1:**
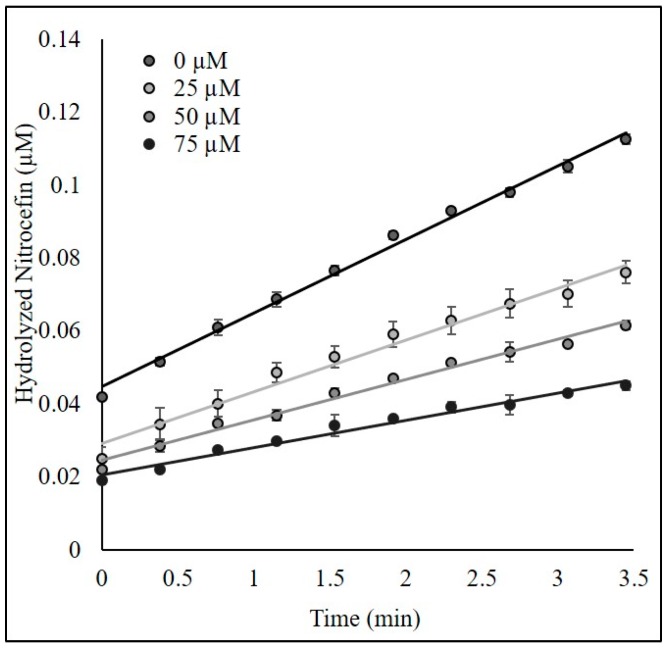
Dose-dependent curve for bis(benzoyl) phosphate after pre-incubation with BlaC.

**Figure 2:**
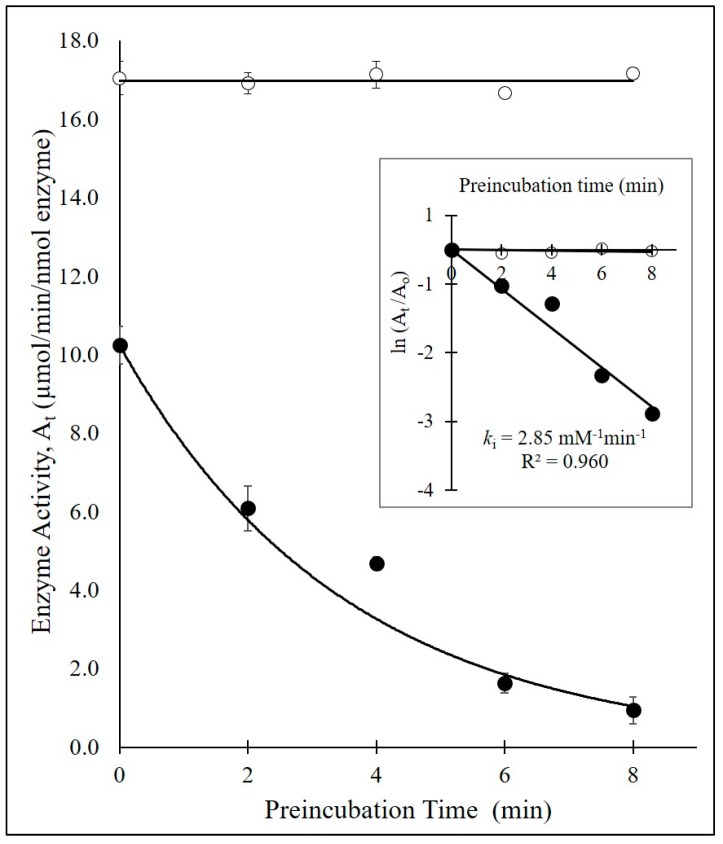
Time-dependent inhibition of BlaC by bis(benzoyl) phosphate (black circles) and BlaC without inhibitor (open circles). Inset figure: replot of the time-dependent residual enzyme activity to determine the bimolecular rate constant *k*_i_ (2.85 mM^-1^·min^-1^) for the inhibition of BlaC by bis(benzoyl) phosphate.

As noted above, Pratt and coworkers demonstrated that acyl phosphonantes and phosphates were inhibitors of β-lactamases. We postulated that this may be due to a time-dependent process involving either acylation or phosphorylation of the active-site Ser-70 reside. To explore the interaction of bis(benzoyl) phosphate with BlaC in more depth, we first confirmed that bis(benzoyl) phosphate inactivated BlaC in a time-dependent manner (Figure 1 and Figure 2). In order to definitively determine this mechanism of inactivation, we sought to compare the crystal structure of BlaC inactivated with the bis(benzoyl) phosphate and the apo form of the enzyme, free of inhibitor.

The crystal structures for both non-phosphoserine BlaC and phosphoserine BlaC were solved using molecular replacement using PDB 2GDN as the search model [6]. The non-phosphoserine BlaC is represented in Figure 2 and Table 1. The phosphoserine structure resolved to 1.52 Å (Table 1). The R-work and R-free for the phosphoserine structure were 0.16 and 0.17, respectively.

**Figure 3:**
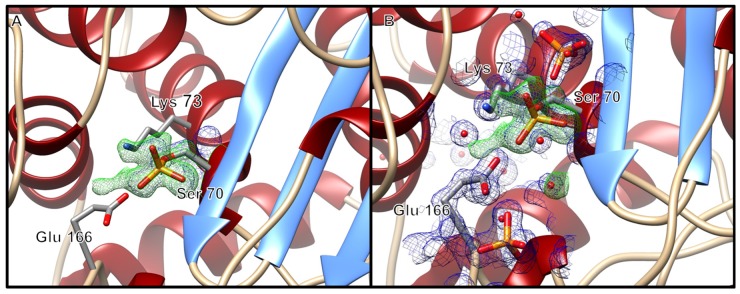
(**A**) Ribbon diagram of the phosphoserine BlaC structure, zoomed in to display electron density about the nucleophilic Ser-70. (**B**) Ribbon diagram of the phosphoserine BlaC structure, with additional phosphate molecules and water molecules with electron density about the active site. The ribbon diagrams have electron density displayed within 3 Å of Ser-70, phosphate molecules and water molecules as a blue mesh at a 1.5 σ contour level for the 2mFo-DFc maps, and the mFo-DFc maps contoured at ±3 σ (green mesh/red mesh). The displayed mFo-Fc map was generated prior to modeling the phosphorylated Ser-70. The figure was generated with UCSF Chimera v1.12.

**Figure 4:**
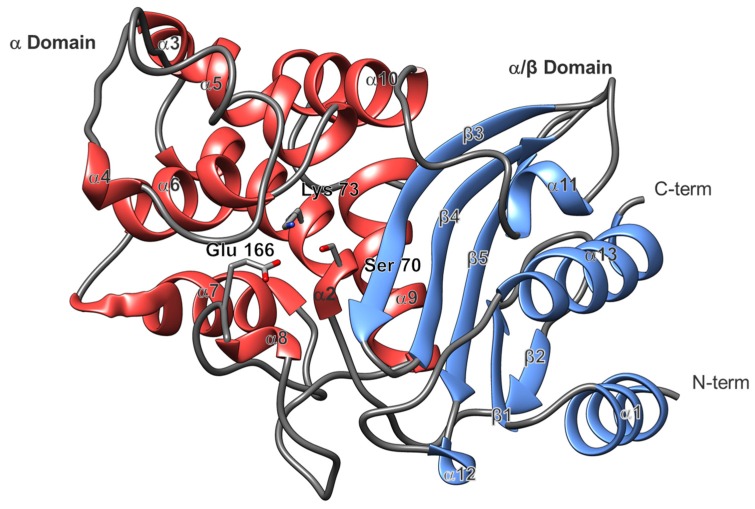
Global BlaC structure. The α-domain is shown in red and the α/β domain is shown in cornflower blue. The active site is positioned toward the center showing the catalytic Ser-70, Lys-73, and Glu-166. Secondary structure numbering corresponds to Figure 5. The figure was generated with UCSF Chimera v1.12.

The phosphoserine crystal was in the P2_1_2_1_2_1_ space group. In the case of the phosphoserine crystal, Ser-70 showed clear density for a phosphoserine having been phosphorylated by the inhibitor bis(benzoyl) phosphate (Figure 3A). Additionally, the phosphoserine BlaC structure contained phosphate molecules near the active site adjacent to the nucleophilic Ser-70 (Figure 3B). In the phosphoserine structure, the distance between the oxygen atom of the phosphorylated Ser-70 and the phosphorus atom of the closest phosphate molecule was 4.5 Å (phosphate pictured above Ser-70 in Figure 3B). The second phosphate molecule pictured at the bottom of Figure 4 was located 9.1 Å from the oxygen atom of the phosphorylated Ser-70. The presence of phosphate molecules adjacent to the active site has previously been reported for BlaC structures as crystallization artifacts, including one in which BlaC was not crystallized with phosphate as the principal component of the crystallization condition and was instead thought to have been a byproduct of purification [16,17]. In addition, even though the previously published BlaC structures contain phosphate molecules near the active site, they do not have a phosphorylated Ser-70. Hereto, the phosphoserine-BlaC (Figure 3) newly demonstrates phosphorylation of the Ser-70 of BlaC.

### 2.2. Overall Structure and Active Site

**Figure 5:**
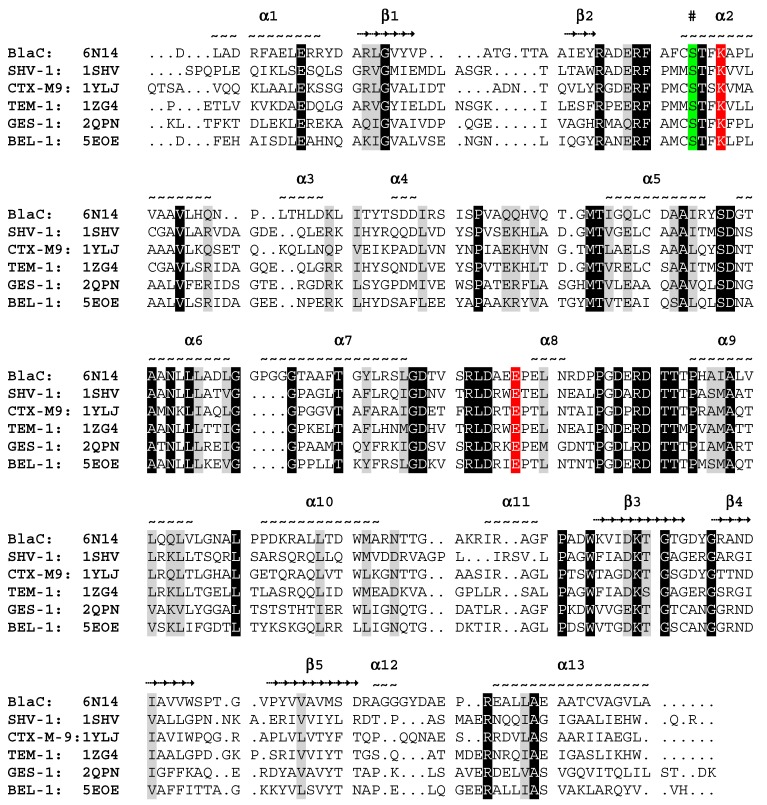
Structural sequence alignment of BlaC with five other class A serine β-lactamases. The protein name and representative PDB ID numbers are listed. Above the sequence, the position of the helices (~) and beta strands (**→**) are indicated and numbered. The nucleophilic Ser-70 is highlighted in green and the catalytic Lys-73 and Glu-166 are highlighted in red.

As previously reported for BlaC and other class A serine β-lactamases such as TEM-1, SHV-1, and the CTXMs, the global fold consists of two domains, the α-domain and the α/β-domain (Figure 4) [8,18,19,20]. Located in the α-domain, adjacent to the α/β-domain, are the catalytic residues Ser-70, Lys-73 and Glu-166 (numbered according to Ambler notation [21]). Structural sequence alignment showed that the primary sequence and the global fold of the α-domain and the α/β-domain were highly conserved throughout class A serine β-lactamases (Figure 5). In the case of BlaC, the α-domain was made up of helices 2 through 11, and the α/β-domain consisted of helices 1, 12, 13, and 14. In addition, the active site exhibits a high degree of conservation for the catalytic residues Ser-70, Lys-73 and Glu-166.

## 3. Discussion

BlaC, a hydrolase (EC 3.5.2.6), cleaves lactam carbon–nitrogen amide bonds rapidly, effectively deactivating β-lactam antibiotics [22,23]. This β-lactam hydrolysis proceeds through an acylation–deacylation reaction. During acylation, Lys-73 acts as a general-base catalyzing the nucleophilic Ser-70 attack of the lactam carbonyl carbon and formation of a tetrahedral intermediate [22,23,24]. The collapse of the tetrahedral intermediate proceeds to the oxyanion-hole stabilized acyl-enzyme adduct. Deacylation proceeds as Glu-166 acts as a general-base and catalyzes a hydrolytic water attack on the carbonyl carbon of the adduct, resulting in the release of the inactive hydrolyzed β-lactam and return of BlaC to its resting state. Due to the efficiency of BlaC, as the main β-lactamase of tuberculosis (TB), to hydrolyze β-lactam antibiotics, the β-lactam class of antibiotics proved ineffective in treating (TB) [2,5,6].

It had been proposed by Pratt and colleagues that phosphate-based compounds could serve as effective inhibitors of β-lactamases [25,26,27,28]. They first demonstrated that a phosphonate monoester inhibited the activity of the class C β-lactamase P99 towards benzylpenicillin [25]. However, upon incubation of the same phosphonate monoester with class A and class D β-lactamases, little inhibition effect was observed. Later work by Rahil and Pratt showed that modifications to the leaving group could expand the inhibitory potency of phosphonates to include class A β-lactamases [26]. They proposed that the mechanism of action for these phosphonates on β-lactamase inhibition directly resulted from nucleophilic Ser-70 phosphorylation. A subsequent crystal structure 1BLH showed a phosphonate inhibitor covalently bound to the Ser-70 of a β-lactamase from *Staphylococcus aureus* as an acyl-enzyme intermediate complex [29]. Building on the promising data by Pratt et al., we proposed using the bis(benzoyl) phosphate to inhibit the activity of the class A β-lactamase BlaC. Upon pre-incubation of the bis(benzoyl) phosphate with BlaC, there was a noted reduction in enzymatic activity as compared to the uninhibited BlaC and time zero preincubation time (Figure 3).

**Figure 6:**
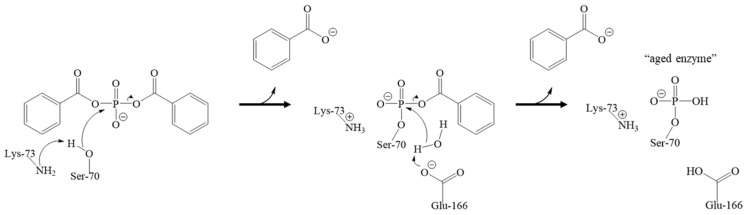
Proposed inactivation mechanism of BlaC. The nucleophilic Ser-70 is deprotonated by Lys-73, which then attacks the phosphorus atom of bis(benzoyl) phosphate, and benzoate leaves. Glu-166 then deprotonates a catalytic water molecule, which attacks the phosphorus to displace the second benzoate, leading to the phosphorylation of Ser-70 and resulting in an aged form of the enzyme.

We surmised that the inactivation of BlaC with bis(benzoyl) phosphate could follow either an acylation or phosphorylation type of mechanism, the latter being consistent with the inactivation of other serine hydrolases with organophosphates [30,31,32]. Even though these compounds contain an acylation site that could lead to an acylated inhibition mechanism, in proposing the phosphorylation mechanism over the acylation mechanism by the bis(benzoyl) phosphate, two equivalents of benzoate would have been released (Figure 6). Consistent with the proposed phosphorylation mechanism, we did not observe an intact inhibitor, nor released benzoates in the active site after the phosphorylation step occurred. Indeed, we hypothesized that a phosphorylation mechanism could be responsible for the mode of inactivation of BlaC with bis(benzoyl) phosphate based on the crystal structure of BlaC, in which Ser-70 is shown to be phosphorylated (Figure 3). Now it should be noted that other groups have demonstrated that the presence of inorganic phosphate in the active site has been shown to reestablish BlaC activity after inactivation by clavulanic acid [17].

It is known that esterase enzymes can be phosphorylated at their nucleophilic serine residues upon exposure to organophosphates through a mechanism of inactivation followed by aging [31,32,33,34]. However, there was no precedence in the literature for the phosphorylation of the active site serine of BlaC, thus it was unknown whether the Ser-70 of BlaC could be irreversibly modified by a phosphorus ligand. We confirmed that when BlaC is incubated with a β-lactamase phosphorylating agent, bis(benzoyl) phosphate, nucleophilic serine phosphorylation occurs based on crystallographic data. In addition, even though the bis(benzoyl) phosphate can theoretically inactivate through an acylation mechanism, the scaffold proceeds through phosphorylation. We propose this occurs via an irreversible time-dependent mechanism based on preliminary inhibitory potency studies (Figure 2 and Figure 3). In theory, the inhibition mechanism involves the Ser-70, Lys-73, Glu-166 and a catalytic water molecule (Figure 6). First, the sidechain amine of Lys-73 deprotonates the hydroxymethyl group of Ser-70. The deprotonated oxyanion of Ser-70 then attacks the phosphorus atom of the bis(benzoyl) phosphate inhibitor and a benzoate group leaves, forming a benzoyl phosphoserine intermediate. Lastly, hydrolysis by a water molecule, deprotonated by the carboxyl sidechain of Glu-166, results in the loss of the second benzoate group, resulting in the aged BlaC being phosphorylated at the nucleophilic Ser-70, not unlike the aged forms of cholinesterase and chymotrypsin inactivated by organophosphorus agents [32,35,36]. However, further studies will need to be conducted to further elucidate the specific amino acid interactions. Due to the novelty of the inactivation of BlaC by the bis(benzoyl) phosphate compound, there is still a vast amount to be investigated.

Presently, the bis(benzoyl) phosphate represents the only phosphorus-containing scaffold that we tested, which inactivated BlaC. Other phosphorus-containing compounds that were tested for inhibition properties included phosphonates and phosphothioesters. A plausible reason why the bis(benzoyl) phosphate scaffold worked over other organophosphate scaffolds might involve the electrophilic nature of the phosphorus center and the pKa of the leaving groups’ conjugate acids (e.g., benzoic acids vs. alcohols and thiols) [30,31,32,37]. This new crystal-structure evidence suggests the inactivation mechanism of BlaC involves the unsubstituted bis(benzoyl) phosphate via nucleophilic Ser-70 phosphorylation, and is expected to aid in the design of future novel β-lactamase inactivators utilizing a phosphorylating scaffold. In the future, these phosphorylating agents can potentially be used concurrently with a β-lactam antibiotic for the treatment of β-lactam antibiotic-resistant bacterial infections.

## 4. Materials and Methods

### 4.1. Expression and Purification

*Escherichia coli* strain BL21 (DE3) transformed with a pET28 plasmid containing the *Mycobacterium tuberculosis* gene *blaC* kindly provided by Dr. Blanchard was used for expression of a truncated version of the β-lactamase protein. The gene was isolated from *Mycobacterium tuberculosis* strain ATCC 25618/H37Rv. The coding region for the first 40 amino acids was removed from the *blaC* gene to aid expression. To express β-lactamase, Luria–Bertani broth was inoculated with transformed *E. coli* cells and incubated at 37 °C until a 0.5 to 0.6 optical density at 600 nm was reached. Cells were then cooled and incubated at 20 °C in a shaker set at 200 rpm for 20 h. After incubation, cells were collected and centrifuged at 3,000 g for 15 min. Pelleted cells were then collected and frozen at −20 ° C until purification.

For purification, frozen cell pellets were suspended in lysis/wash buffer containing 20 mM Tris, 300 mM NaCl, and 20 mM imidazole at pH 8.5. The resuspended cells were sonicated with a Branson Sonifier^TM^ 450 (Branseon Ultrasonics, Danbury, CT, U.S.A.) to release soluble protein and then centrifuged at 15,000 g for 30 min. The supernatant was added to a Ni-NTA column (Bio-Rad, CA, U.S.A.). The Ni-NTA was then washed with 10 column volumes of the lysis/wash buffer. β-lactamase was eluted from the Ni-NTA column with a buffer containing 20 mM Tris, 300 mM NaCl, and 250 mM imidazole at pH 8.5. Next, the eluted β-lactamase was buffer exchanged into buffer A (20 mM Tris pH 8.5, 2 mM DTT, 5% glycerol) for purification on a GE ÄKTA pure FPLC system (GE Healthcare, PA, U.S.A.) connected to a Resource^TM^ Q (GE Healthcare, PA, U.S.A.) column. β-lactamase BlaC was eluted from the Resource^TM^ Q column by running a gradient of buffer A to buffer B (20 mM Tris pH 8.5, 2M NaCl, 5% glycerol). Purified β-lactamase fractions were collected for future use based on SDS-PAGE.

### 4.2. Synthesis of the Bis(Benzoyl) Phosphate

Pyridine (2 mL) was added to a 20 mL scintillation vial containing 20−25 glass beads (5 mm) and 1 g (7.04 mmol) sodium phosphate (dibasic, anhydrous). The slurry was vortexed for 2−5 min before the addition of the acyl chloride derivative (15.49 mmol, 2.2 equivalences). After the addition, the reaction vial was continuously vortexed for 1 hr. Afterwards, a solution of 10% HCl_(aq)_: ethyl acetate (1:1, *v*/*v*) was added to the crude reaction mixture then transferred into a separatory funnel. The aqueous layer was extracted 3× with ethyl acetate and the organic layers were pooled together then washed 3× with saturated sodium chloride solution. The precipitant which formed during each brine wash was collected and placed on a vacuum to remove residual water. The crude solid was further purified by reverse-phase (RP) C18 column chromatography (Flash Biotage, Sweden) using a gradient mobile phase 50%−70% acetonitrile: water (*v*/*v*). White solid in >99% isolated yield. ^1^H NMR (400 MHz, DMSO-d6) δ: 7.97 (t, J = 1.6 Hz, 2H), 7.67 (d, J = 7.4 Hz, 1H), 7.54 (t, J = 7.9 Hz, 2H). ^13^C NMR (101 MHz, DMSO-d6) δ 162.30, 162.21, 133.66, 130.38, 130.31, 129.78, 128.82. ^31^P NMR (122 MHz, DMSO-d6) δ -6.00 (triphenyl phosphine std, s), -17.33(s). HRMS (MALDI) m/z calculated for C_14_H_11_O_6_P [M + Na] 329.0191; found [M + Na] 329.0186 (± 1.6 ppm).

### 4.3. Time-Dependent Inhibition of BlaC

To determine the bimolecular rate constant for the time-dependent inhibition of BlaC by bis(benzoyl) phosphate, BlaC was incubated with bis(benzoyl) phosphate in the absence of the substrate (nitrocefin) for increasing periods of time (0–8 min). Progress curves for the hydrolysis of nitrocefin to provide the residual enzyme activity A_t_ were acquired using A Fluostar Omega Microplate Reader running Omega software version 1.02 and Mars Data Analysis Software Program version 1.10 (BMG Labtech. For each pre-incubation time point, 75 µL of 10 nM BlaC was added to replicate wells with 100 µM of bis(benzoyl) phosphate in 100 mM PIPES Buffer pH = 7.4 at 37 °C. After pre-incubation for each specified time point, the residual enzymatic activity was determined by the addition of 75 µL of 100 µM nitrocefin. The rate of product formation was determined by measuring the absorbance at 482 nm (ε = 17, 400 M^-1^·cm^-1^) over the course of 8.7 min to generate an enzyme activity A_t_ for each time point of preincubation (µmol/min/nmol enzyme).

### 4.4. Protein Crystallization and Structure Determination

β-lactamase crystals were grown using the hanging-drop vapor diffusion method at 4 °C. A modified crystallization solution of 0.1 M HEPES pH 7.4, 2.25 M ammonium phosphate monobasic was used [8]. Non-phosphoserine crystals were grown by making drops containing 1 part purified β-lactamase at 15 mg/mL in 20 mM Tris pH 8.5, 150 mM NaCl with 1-part crystallization solution and equilibrating against the same reservoir. For the β-lactamase Ser-70 phosphorylated crystals, drops contained 1 part purified β-lactamase at 15 mg/mL (0.46 mM) and the inhibitor at 1 mM bis(benzoyl) phosphate in 20 mM Tris pH 8.5, 150 mM NaCl. After the inhibitor and β-lactamase were gently mixed, the mixed protein and inhibitor solution was directly mixed without preincubation with 1-part crystallization solution and equilibrated against the reservoir solution. Crystals typically formed within two weeks. Prior to data collection, crystals were flash frozen using liquid nitrogen and the cryoprotectant 0.1 M HEPES pH 7.4, 2.25 M ammonium phosphate monobasic, and 20% glycerol. Crystal data were collected at Advanced Light Source (ALS) beamlines 5.0.1 and 8.2.1. Diffraction data processing was done with the HKL2000 software package [38]. Molecular replacement was done using PHENIX Phaser and a previously solved β-lactamase (BlaC) with the same sequence (PDB: 2GDN) [8,39]. PHENIX and Coot were used for refinement and modeling [39,40].

### 4.5. Sequence Alignment

Structural comparison and sequence alignment of serine β-lactamases were conducted using UCSF Chimera software and the TM-align server to calculate c-alpha RMSD and TM-score [41,42]. Sequence identity and similarity were calculated using the LALIGN tool with alignment set for global without gap open penalty, available on the SIB ExPASy Bioformatics Resources Portal website. Serine β-lactamases with deposited crystal structures were found using the Beta-Lactamase Data Base and the corresponding models were downloaded from the PDB [43,44]. The MatchMaker tool in Chimera was used to generate the structural sequence alignment and crystal structure superposition [41]. The sequence alignment figure was generated with BioEdit and manually annotated [45].

## Figures and Tables

**Table 1:** Crystal data table, parentheses refer to highest resolution shell.

**Data collection**	**BlaC - phosphoserine**
PDB ID	6N14
Space group	P2_1_2_1_2_1_
Cell dimensions	
(a, b, c) (Å)	49.84, 68.04, 75.45
Molecules per asymetric unit	1
Resolution (Å)	28.10−1.52
Wavelength (Å)	1.00
R_sym_	0.099 (0.816)
I/σ	17.6 (1.92)
CC_1/2_	0.992 (0.623)
Completeness	99.75 (97.88)
Redundancy	6.8 (4.7)
**Refinement**	
Resolution (Å)	28.10−1.52
Unique reflections	39,978 (3,879)
R_work_/R_free_	0.155/0.169
RMSD	
RMSD bonds (Å)	0.003
RMSD angles (Å)	0.745
Ramachandrans (%)	
Favored	98
Outliers	0
Number of atoms	2,322
Protein and ligand	1,999
Water	323
